# Supplementary material for: Using N-doped Carbon Dots Prepared Rapidly by Microwave Digestion as Nanoprobes and Nanocatalysts for Fluorescence Determination of Ultratrace Isocarbophos with Label-Free Aptamers
Source: Nanomaterials (Basel). 2019 Feb 7;9(2):223. doi: 10.3390/nano9020223 (PMC6409902; doi:10.3390/nano9020223)
Supplement: Supplementary file 1 [file nanomaterials-09-00223-s001.pdf]

Using N-doped Carbon Dots Prepared Rapidly by Microwave Digestion as Nanoprobes and Nanocatalysts for Fluorescence Determination of Ultratrace Isocarbophos with Label-Free Aptamers

Xin Li, Xin Jiang, Qingye Liu*, Aihui Liang, Zhiliang Jiang*

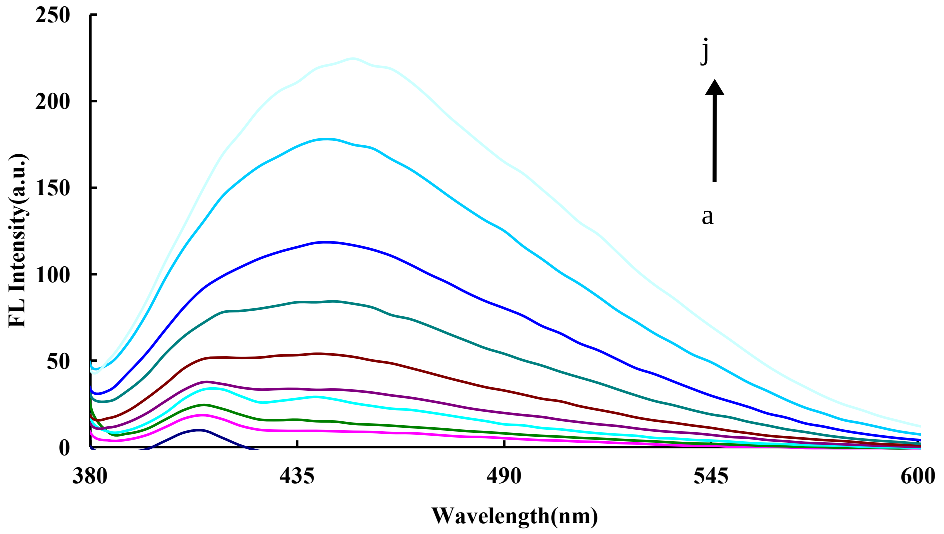

Figure S1. Fluorescence spectrum of CDg. a: 0 mg/L CDg;b: 1.41 mg/L CDg; c: 2.77 mg/LCDg;d: 5.44 mg/LCDg;e: 10.88 mg/LCDg;f: 22.21 mg/LCDg;g: 44.43 mg/LCDg;h: 86.13 mg/LCDg;i: 176.8 mg/LCDg;j: 353.6 mg/LCDg

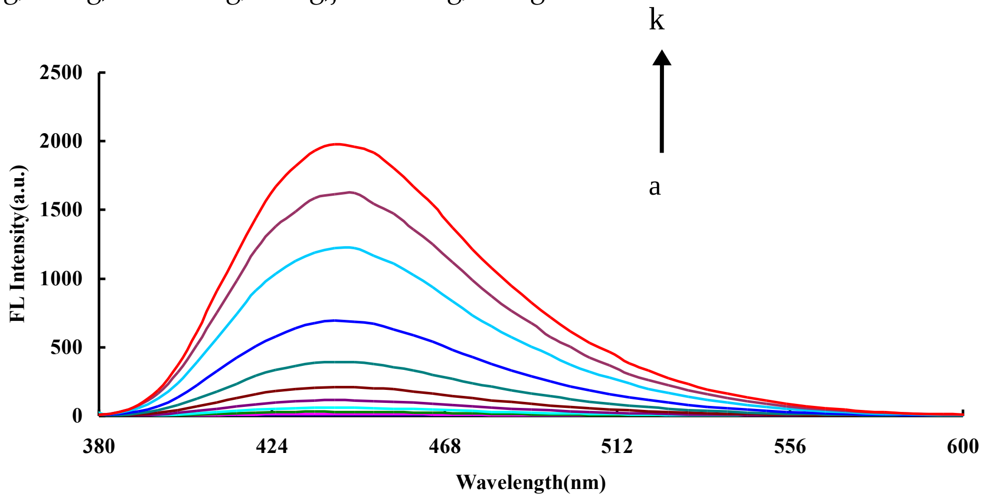

Figure S2. Fluorescence spectra of CDs. a: 0 mg/L CDs; b: 6.24 mg/L CDs; c: 12.48 mg/L CDs; d: 24.96 mg/L CDs; e: 50.44 mg/L CDs; f: 101.4 mg/L CDs; g: 202.8 mg/L CDs; h: 406.12 mg/L CDs; i: 812.24 mg/L CDs; j: 1625 mg/L CDs; k: 3250 mg/L CDs

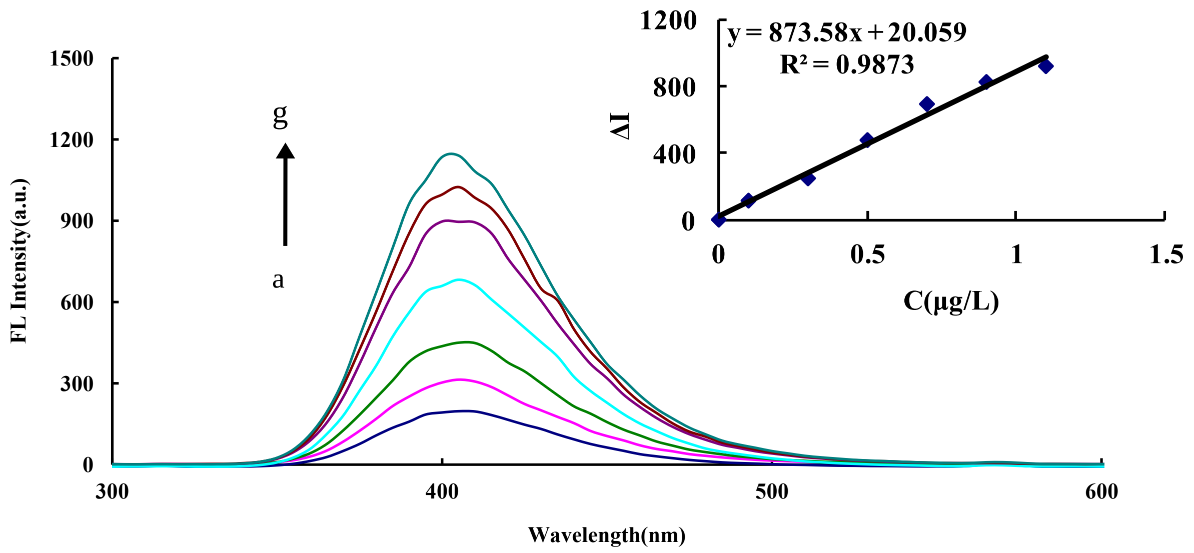

Figure S3. Fluorescence spectrum of the Apt–IPS–CDg–H_2O_2–TMB–HAc–NaAc system. a: 31 nmol/L Apt + 0.113 mg/L CDg + 0.053 mmol/L H_2O_2 + 0.017 mmol/L TMB + 0.13 mmol/L pH 3.6 HAc–NaAc; b: a + 0.1 μg/L IPS; c: a + 0.3μg/L IPS; d: a + 0.5μg/L IPS; e: a + 0.7μg/L IPS; f: a + 0.9μg/L IPS; g: a + 1.1μg/L IPS

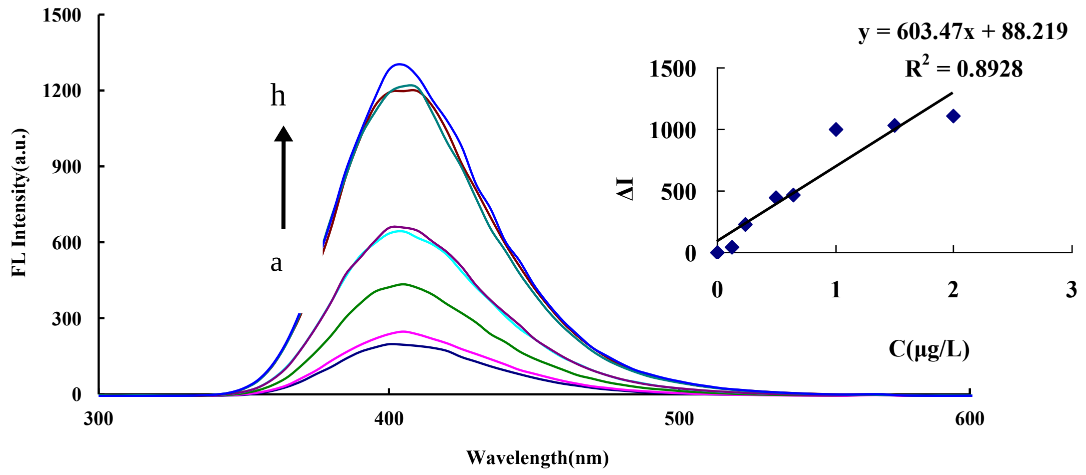

Figure S4. Fluorescence spectrum of the Apt–IPS–CDs–H_2O_2–TMB–HAc–NaAc system. a: 31 nmol/L Apt + 0.26 mg/L CDs + 0.053 mmol/L H_2O_2 + 0.017 mmol/L TMB + 0.13 mmol/L pH 3.6 HAc–NaAc; b: a + 0.12 μg/L IPS; c: a + 0.24μg/L IPS; d: a + 0.5μg/L IPS; e: a + 0.64μg/L IPS; f: a + 1.0μg/L IPS; g: a + 1.5μg/L IPS; h: a + 2μg/L IPS

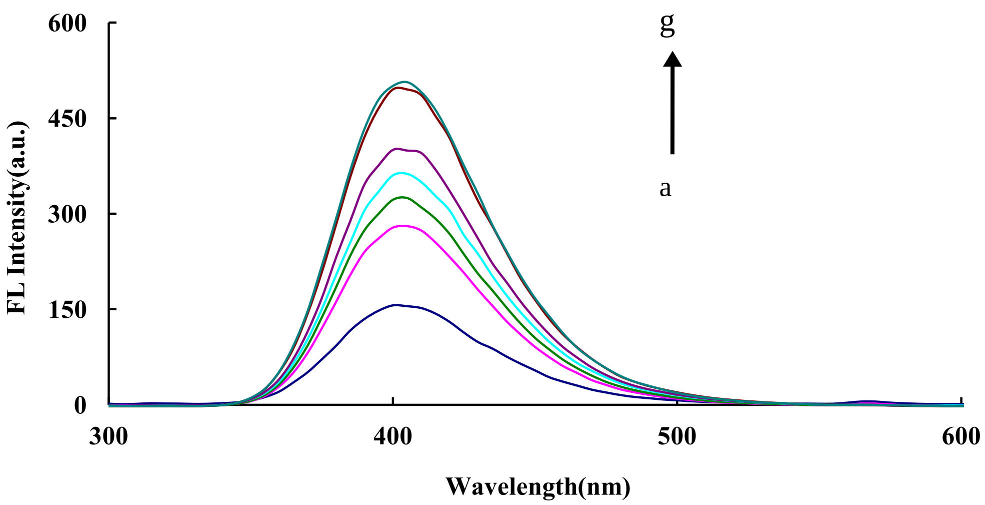

Figure S5. Fluorescence spectrum of the CDg–H_2O_2–TMB–HAc–NaAc system. a: 0.13 mmol/L H_2O_2 + 33 μmol/L TMB + 0.13 mmol/L pH 3.6 HAc–NaAc; b: a + 0.023 mg/L CDg; c: a + 0.061 mg/L CDg; d: a + 0.076 mg/L CDg; e: a + 0.113 mg/L CDg; f: a + 0.189 mg/L CDg; g: a + 0.227 mg/L CDg

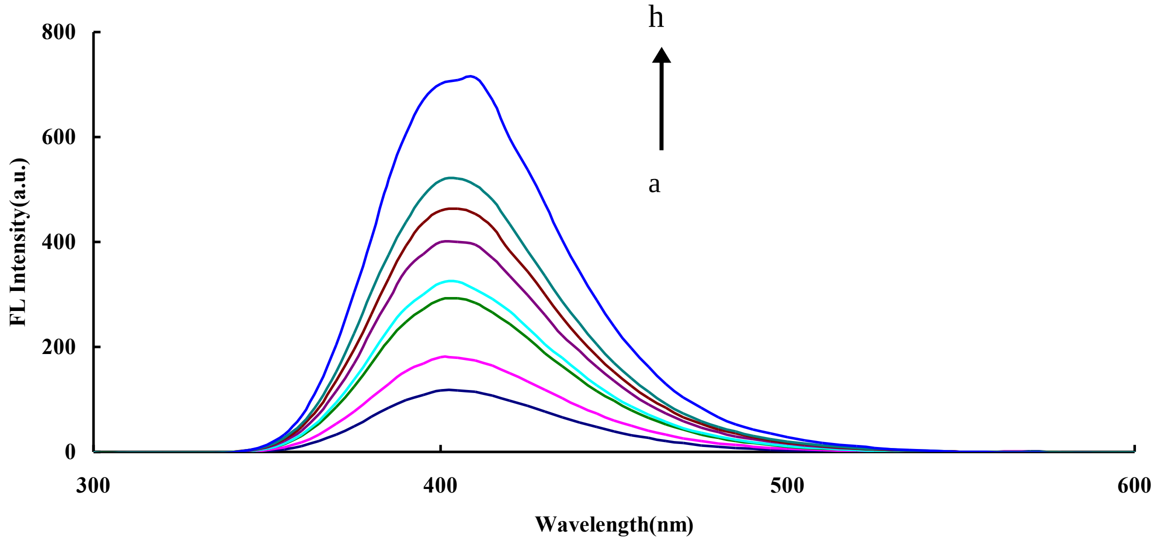

Figure S6. Fluorescence spectrum of the CDs–H_2O_2–TMB–HAc–NaAc system. a: 0.13 mmol/L H_2O_2 + 33 μmol/L TMB + 0.13 mmol/L pH 3.6 HAc–NaAc; b: a + 0.009 mg/L CDs; c: a + 0.043 mg/L CDs; d: a + 0.086mg/L CDs; e: a + 0.13 mg/L CDs; f: a + 0.173 mg/L CDs; g: a + 0.26 mg/L CDs; h: a + 0.347 mg/L CDs

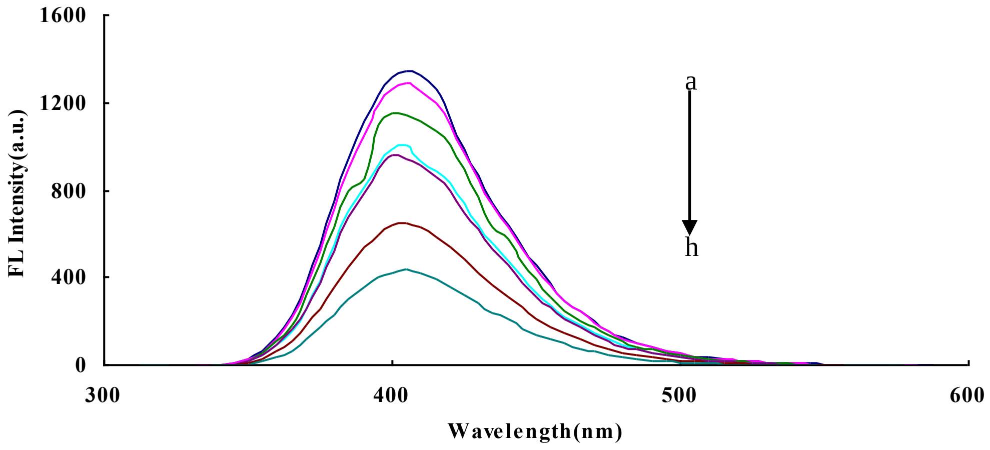

Figure S7. Fluorescence spectrum of the Apt–CDg–H_2O_2–TMB–HAc–NaAc system. a: 0.227 mg/LCDg + 0.053 mmol/L H_2O_2 + 0.017 mmol/L TMB + 0.13 mmol/L pH 3.6 HAc–NaAc; b: a + 5.17 nmol/L Apt; c: a + 10.33 nmol/L Apt; d: a + 15.5 nmol/L Apt; e: a + 20.67 nmol/L Apt IPS; f: a + 25.83 nmol/L Apt; g: a + 31 nmol/L Apt; h: a + 25.83 nmol/L Apt.

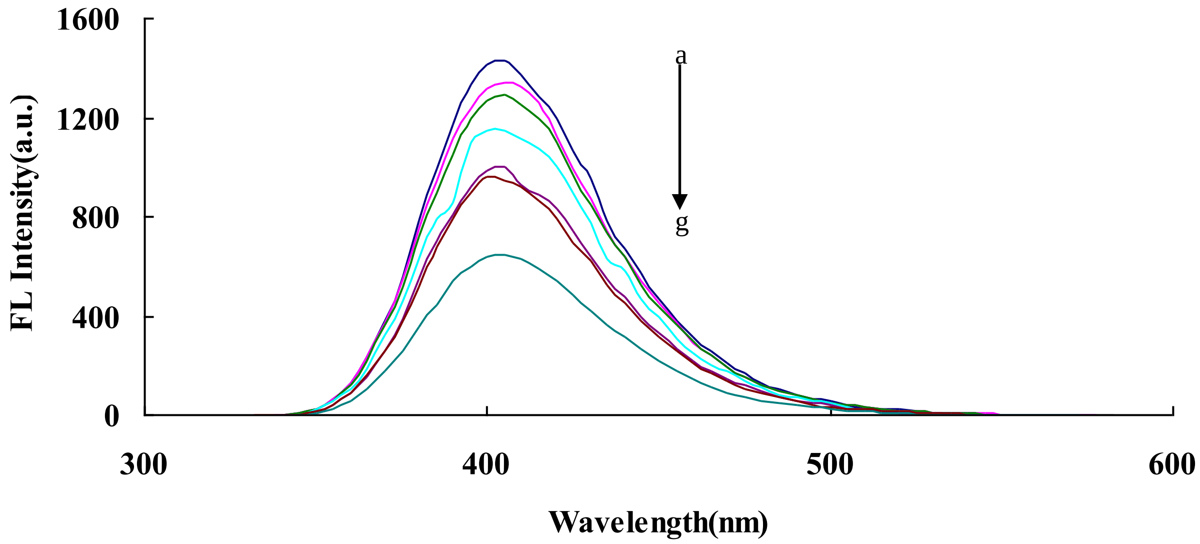

Figure S8. Fluorescence spectrum of the Apt–CDs–H_2O_2–TMB–HAc–NaAc system. a: 0.347 mg/LCDs + 0.053 mmol/L H_2O_2 + 0.017 mmol/L TMB + 0.13 mmol/L pH 3.6 HAc–NaAc; b: a + 5.17 nmol/L Apt; c: a + 7.23 nmol/L Apt; d: a + 15.5 nmol/L Apt; e: a + 20.67 nmol/L Apt IPS; f: a + 25.83 nmol/L Apt; g: a + 31 nmol/L Apt.

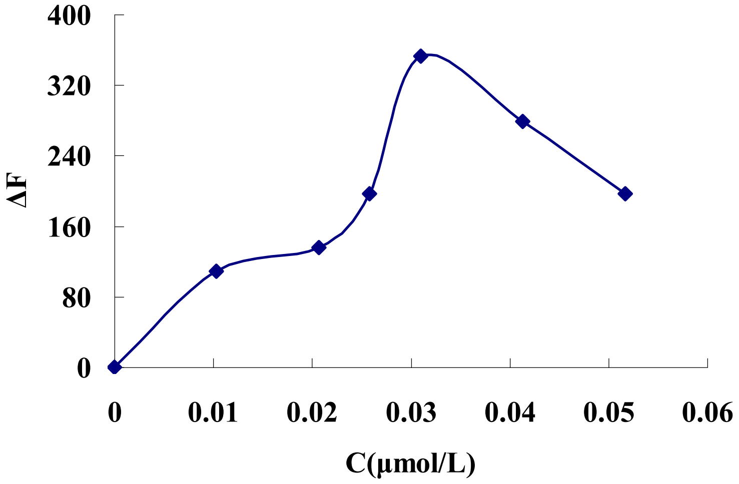

Figure S9. Effect of Apt concentration on system ΔF. Apt + 0.2 μg/L IPS + 3.33 μg/L CD, a + 0.08 mmol/L H_2O_2 + 0.025 mmol/L TMB + 0.13 mmol/L pH 5.6 HAc–NaAc.

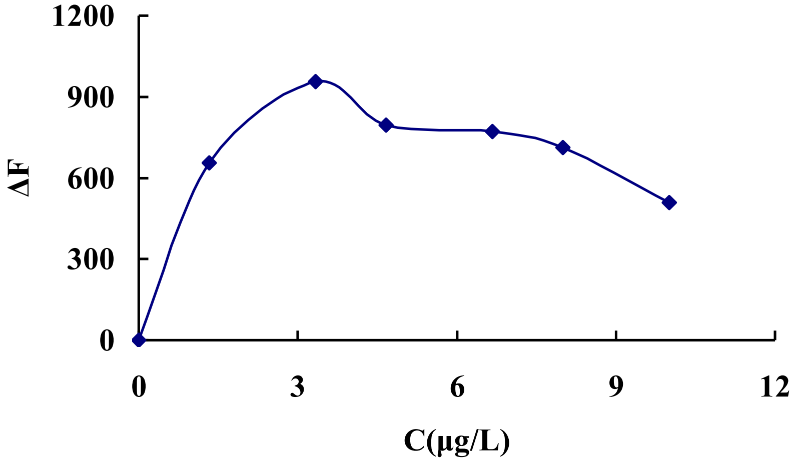

Figure S10. Effect of CD_N concentration on system ΔF. 0.031 μmol/L Apt + 0.2 μg/L IPS + 0.08 mmol/L H_2O_2 + 0.025 mmol/L TMB + 0.13 mmol/L pH 3.6 HAc–NaAc.

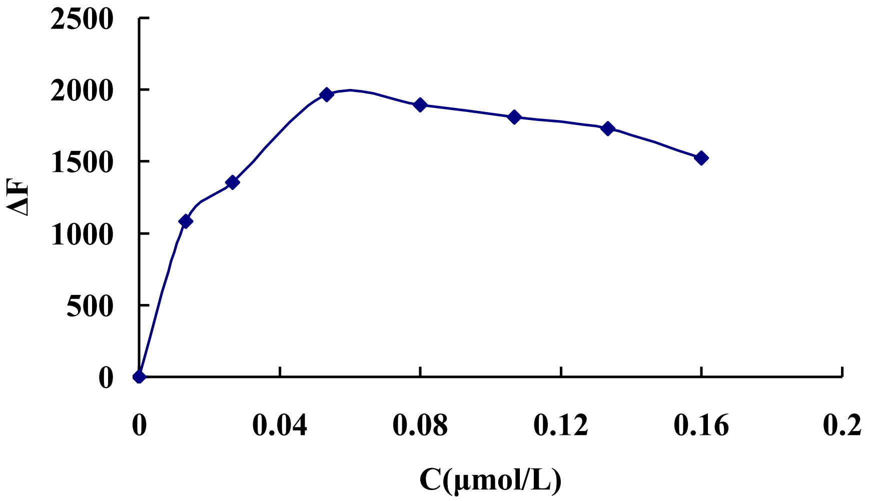

Figure S11. Effect of H_2O_2 concentration on system ΔF. 0.031 μmol/L Apt + 0.2 μg/L IPS + 3.33 μg/L CD, a + H_2O_2 + 33 μmol/L TMB + 0.13 mmol/L pH 3.6 HAc–NaAc.

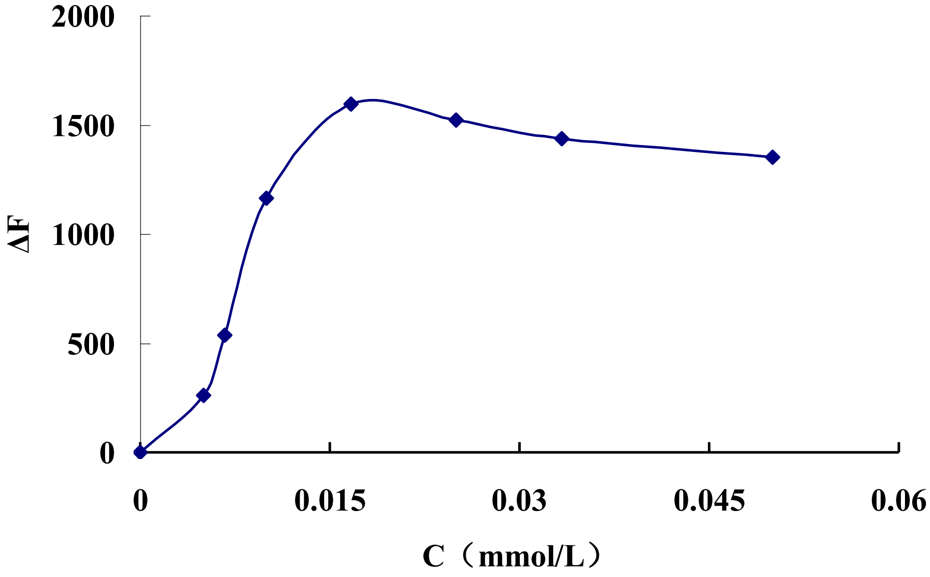

Figure S12. Effect of TMB concentration on system ΔF. 0.031 μmol/L Apt + 0.2 μg/L IPS + 3.33 μg/L CD_N + 0.053 mmol/L H_2O_2 + TMB+0.13 mmol/L pH 3.6 HAc–NaAc

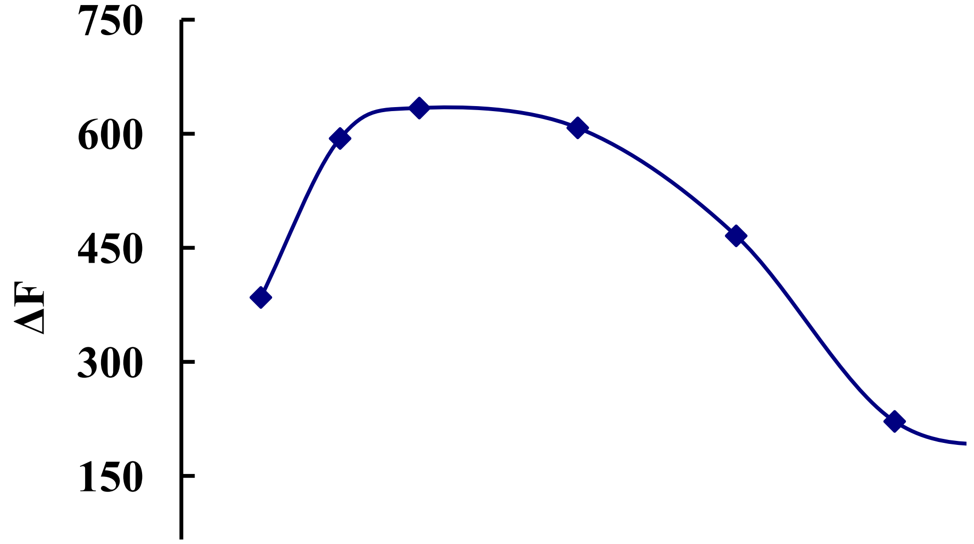

Figure S13. Effect of pH on system ΔF. 0.031 μmol/L Apt + 0.2 μg/L IPS + 3.33 μg/L CD_N + 0.053 mmol/L H_2O_2 + 0.017 mmol/L TMB.

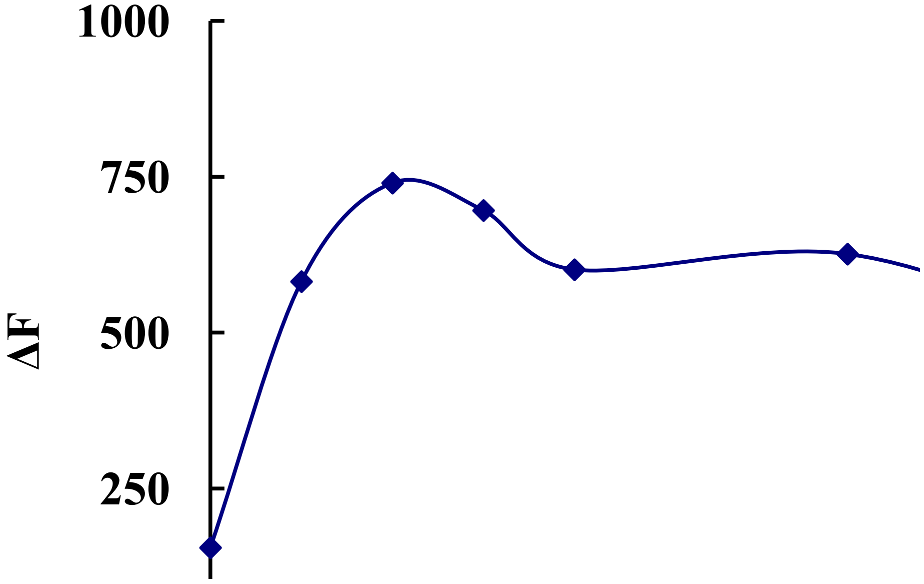

Figure S14. Effect of HAc–NaAc buffer solution on system ΔF. 0.031 μmol/L Apt + 0.2 μg/L IPS + 3.33 μg/L CD_N + 0.053 mmol/L H_2O_2 + 0.017 mmol/L TMB + pH 3.6 HAc–NaAc.

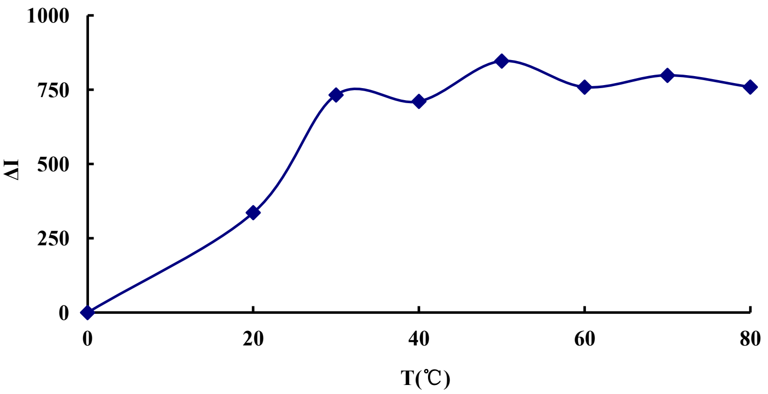

Figure S15. Effect of temperature on the ΔF of the system. 0.031 μmol/L Apt + 0.2 μg/L IPS + 3.33 μg/L CD_N + 0.053 mmol/L H_2O_2 + 0.017 mmol/L TMB + 0.13 mmol/L pH 3.6 HAc–NaAc

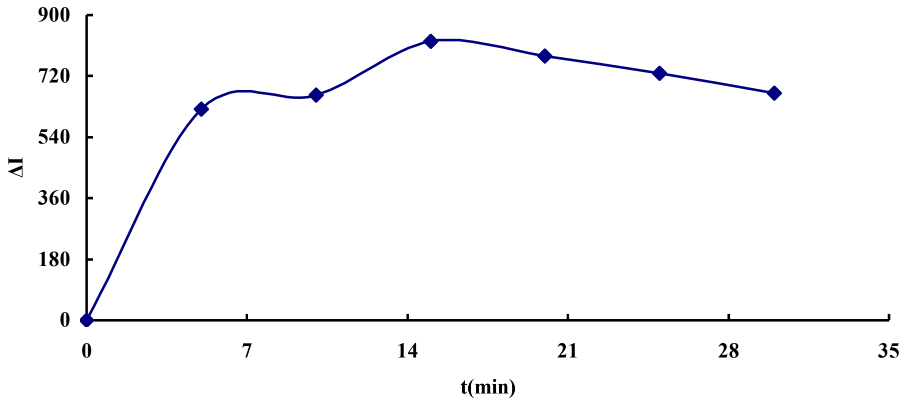

Figure S16. Effect of time on system ΔF. 0.031 μmol/L Apt + 0.2 μg/L IPS + 3.33 μg/L CD_N + 0.053 mmol/L H_2O_2 + 0.017 mmol/L TMB + 0.13 mmol/L pH 3.6 HAc–NaAc.

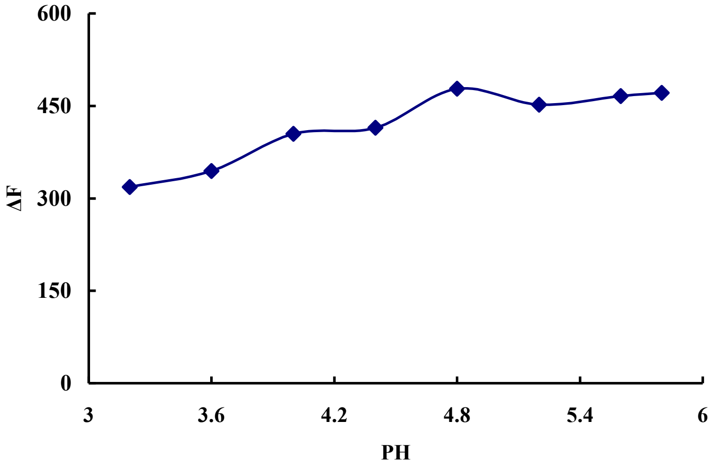

Figure S17. Effect of pH on system ΔF. 0.21 μmol/L Apt + 1.5 μg/L IPS + 7.3 mg/L CD_N.

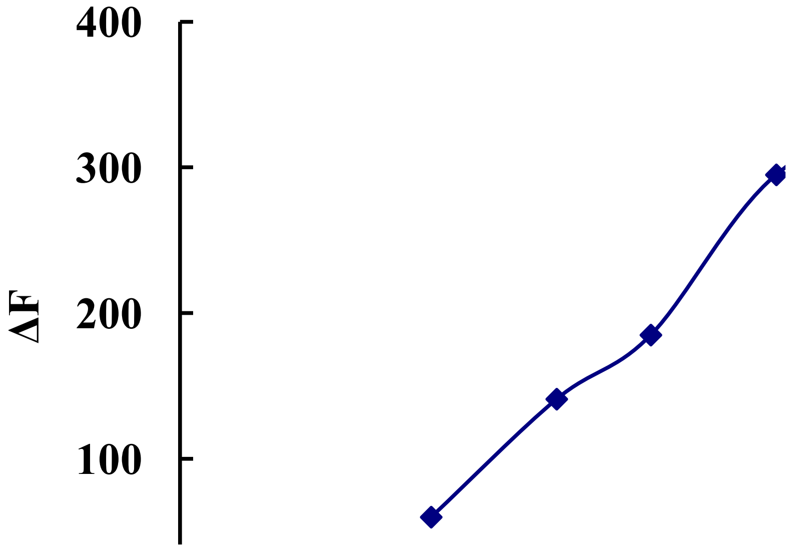

Figure S18. Effect of pH on system ΔF. 0.21 μmol/L Apt + 1.5 μg/L IPS + 7.3 mg/L CD_N.

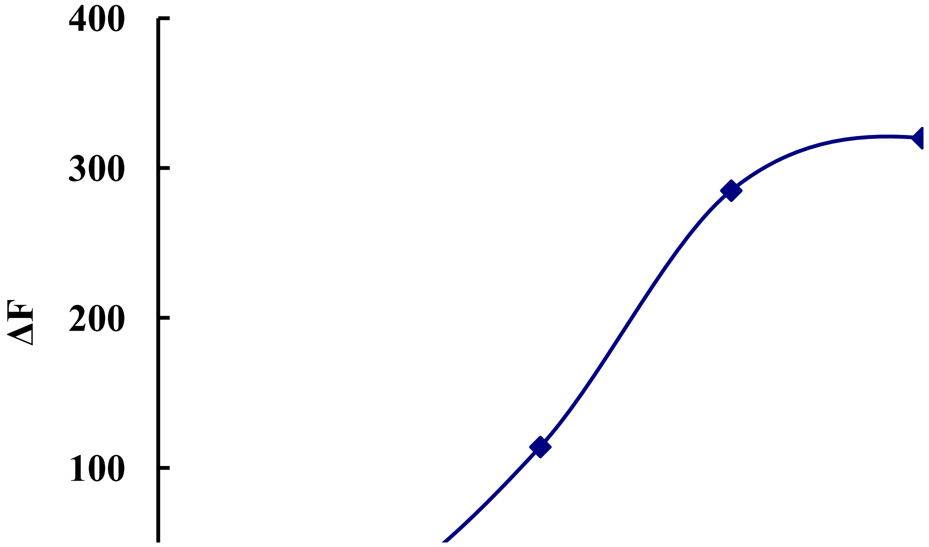

Figure S19. Buffer solution concentration on the system **ΔF.** 0.21 μmol/L Apt + 1.5 μg/L IPS + 7.3 mg/L CD_N.

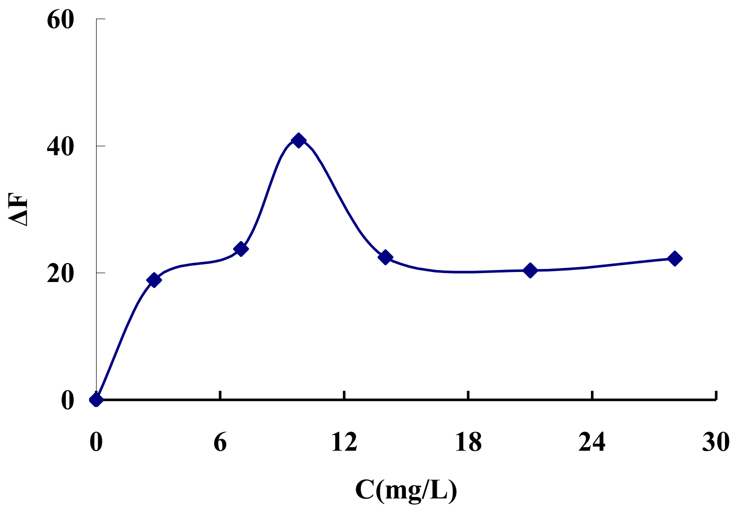

Figure S20. Effect of CD_N concentration on system ΔF. 0.21 μmol/L Apt + 1.5 μg/L IPS + CD_N.

Table S1. Effect of CES on the nanocatalytic fluorescence measurement of IPS.

CES	Tolerance (C_{CES}/C_{IPS})	Error (%)	CES	Tolerance (C_{CES}/C_{IPS})	Error (%)
Zn^{2+}	1000	5.3	Fe^{3+}	250	–8.1
Ca^{2+}	1000	–2.0	Bi^{3+}	250	–5.7
Ni^{2+}	1000	2.5	Cu^{2+}	100	–2.7
Mn^{2+}	500	6.4	Pb^{2+}	250	6.4
Co^{2+}	1000	0.5	Al^{3+}	500	3.9
NH_4Cl	250	–2.3	Cr^{6+}	100	–3.8
CO_3^{2-}	500	5.8	Fe^{2+}	100	8.6
Ba^{2+}	1000	0.3	Hg^{2+}	100	–4.3
Mg^{2+}	1000	4.2	glyphosate	1000	5.5
K^+	1000	6.7	tributylphos phine	1000	–1.9
HCO_3^-	500	7.1	profenofos	1000	3.1
NO_2^-	500	2.9	DNA_F[a]	300	4.5

[a] The sequence of DNA_F is CAT CTC TTC TCC GAG CCG.

Table S2. Effect of CES

CES	Tolerance (C_{CES}/C_{IPS})	Error (%)	CES	Tolerance (C_{CES}/C_{IPS})	Error (%)
Zn^{2+}	1000	3.9	NO_2^-	500	3.8
Ca^{2+}	1000	–6.1	Fe^{3+}	250	–6.5
Ni^{2+}	1000	5.7	Bi^{3+}	250	–2.9
Mn^{2+}	500	2.6	Cu^{2+}	100	–3.7
Cr^{6+}	250	–4.8	Pb^{2+}	250	5.4
Co^{2+}	250	3.5	Al^{3+}	250	4.3
NH_4Cl	500	–2.7	Fe^{2+}	100	–7.4
CO_3^{2-}	500	3.7	Hg^{2+}	100	–8.1
Ba^{2+}	1000	0.5	glyphosate	1000	8.5
Mg^{2+}	1000	2.9	tributylphos phine	500	–3.1
K^+	1000	5.8	profenofos	500	6.2
HCO_3^-	500	4.9	DNA_F	100	5.8

Table S3. Determination results of IPS in water samples.

**Sample**	**Measured value (μg/L, *n* = 5)**	**Added (μg/L)**	**Found (μg/L)**	**Recovery (%)**	**RSD (%)**	**Ref. results (μg/L)**
Domestic sewage	—	0.1	0.0985	98.5		—
Farmland water	0.08	0.1	0.181	101	2.4	0.637
Pond water	0.05	0.1	0.154	104	2.9	0.34
